# Paraneoplastic Autoimmune Hemolytic Anemia Secondary to a Gastric Duplication Cyst in a 15-Month-Old Girl: A Case Report and Review of the Literature

**DOI:** 10.7759/cureus.75949

**Published:** 2024-12-18

**Authors:** Archana Sharma, Richard Drachtman, Alysta M Paneque, Vineeta Maddali

**Affiliations:** 1 Pediatric Hematology, Robert Wood Johnson University Hospital, New Brunswick, USA; 2 Medicine, Robert Wood Johnson Medical School, New Brunswick, USA; 3 Pediatrics, Mount Sinai Hospital, New York, USA

**Keywords:** adenomyomatosis, gastric duplication, malignancy, paraneoplastic autoimmune hemolytic anemia, pediatric

## Abstract

Gastric duplication is a rare congenital disorder of the alimentary tract, and anemia is a rare presenting clinical sign of gastric duplication cysts (GDC). There are two reported cases of autoimmune hemolytic anemia as a paraneoplastic presentation of gastric duplication in the adult population, but there have not yet been any reported cases of this in childhood. We report the first case in the pediatric patient population: the case of GDC presenting as autoimmune hemolytic anemia in a 15-month-old girl.

## Introduction

Gastric duplication is a rare congenital disorder of the alimentary tract that has been reported to occur anywhere from the mouth to the anus. Gastric duplication cysts (GDCs) are defined as spherical or tubular structures with a mucosal-lined muscular layer similar to the adjacent gastrointestinal tract [1]. Though cysts are most likely found in the ileal region, GDCs, specifically account for 2-9% of all gastric duplication cysts, found predominantly along the greater curvature [2]. The most widely held theories for the development of gastric duplication include partial twinning, a phenomenon stating that organs can be doubled as a result of abnormal twinning. Additional theories include defects in notochord development and incomplete bowel recanalization, persistent embryonic diverticula, and intrauterine strokes [3].

Most GDCs are initially benign in children. These cysts may exist without symptoms, growing slowly until obstruction arises due to mass effect [4]. The clinical manifestations of gastric duplications specifically range from asymptomatic to palpable abdominal mass associated with signs of gastric outlet obstruction, vomiting, abdominal distension, melena, chronic pancreatitis, and weight loss/failure to thrive [5]. These signs are nonspecific and are commonly mistaken for other, more commonplace gastrointestinal pathologies [3]. However, the treatment of GDC is surgical due to the malignant potential of the tissue. There are no reported pre-operative diagnostic tests that can determine whether the cyst is benign or malignant [4]. Therefore, without surgery, paraneoplastic manifestations of the cyst may develop [6].

Anemia is a rare presenting clinical sign of GDC. Autoimmune hemolytic anemia as a paraneoplastic phenomenon of gastric duplication has been reported twice in the adult population [7,8]. We report the first case of autoimmune hemolytic anemia due to a GDC in a child.

## Case presentation

We report a case of a 15-month-old female born at 37 weeks gestation via cesarean section for a twin pregnancy without prenatal or perinatal complications. The patient was breast-fed, and growth and development were tracked appropriately. Her identical twin sister was in good health. The patient’s presentation is unique in that her anemia was incidentally discovered at the time of lead screening at 11 months of age. Initial evaluation done prior to consultation of our department included a complete blood count (CBC) that revealed a hemoglobin of 5.4 g/dL (normal range: 10.5-13.5 g/dL), a hematocrit of 19.9% (normal range: 33-49%), a white blood cell (WBC) count of 13,800 mm^3^ (normal range: 6,000-17,000 cells per mm^3^), platelets of 501,000 mcL (normal range: 150,000-450,000 platelets per mcL), mean corpuscular volume (MCV) of 88 fl (normal: 70-86 fL), and MCHC of 27.1 g/dL (normal: 30-36 g/dL). Further iron studies revealed an iron level of 29 mcg/dL (normal: 30-70 mcg), total iron-binding capacity of 244 mcg/dL (normal range: 18-126 mcg/dL), transferrin saturation of 8.4% (normal: 22-39%), and a ferritin of 3.6 ng/mL (normal: 12-71 ng/mL). A comprehensive metabolic panel (CMP) revealed a bilirubin of 0.18 mg/dL (normal: 0.00-0.40). Despite a slightly elevated MCV, the patient was treated for severe iron deficiency anemia. Over the subsequent year, the patient was intermittently treated with oral iron supplementation of 3 mg/kg/day, with only a partial response following an initial transfusion.

Our department was consulted to evaluate the patient at 15 months of age, after initiation of iron supplementation. At this time, the patient was found to have a hemoglobin of 6 g/dL, a significant corrected reticulocyte count of 14.33% (normal: 0.6-2.6%), elevated MCV of 88.1 fL, and lactate dehydrogenase (LDH) of 427 IU/L (normal: 125-220 IU/L), and a physical exam significant for marked pallor. A direct Coombs test was negative. RBC morphology and hemoglobin electrophoresis were both normal. There was no splenomegaly or jaundice on the physical exam. The lack of response to iron transfusion and consistently elevated MCV, which is typically low in iron deficiency anemia, raised concern that the patient did not have severe iron deficiency anemia. Instead, the combination of low hemoglobin, low hematocrit, elevated LDH, and significant reticulocytosis were concerning for hemolytic anemia. The patient was subsequently admitted to our institution for transfusion of packed red blood cells and further evaluation of her severe and persistent anemia. During her hospital course, the patient was found to have a low albumin of 2.9 g/dL (normal: 3.5-5.0 g/dL), which, when combined with her persistently low iron level, prompted evaluation for a primary gastrointestinal etiology.

**Figure 1:**
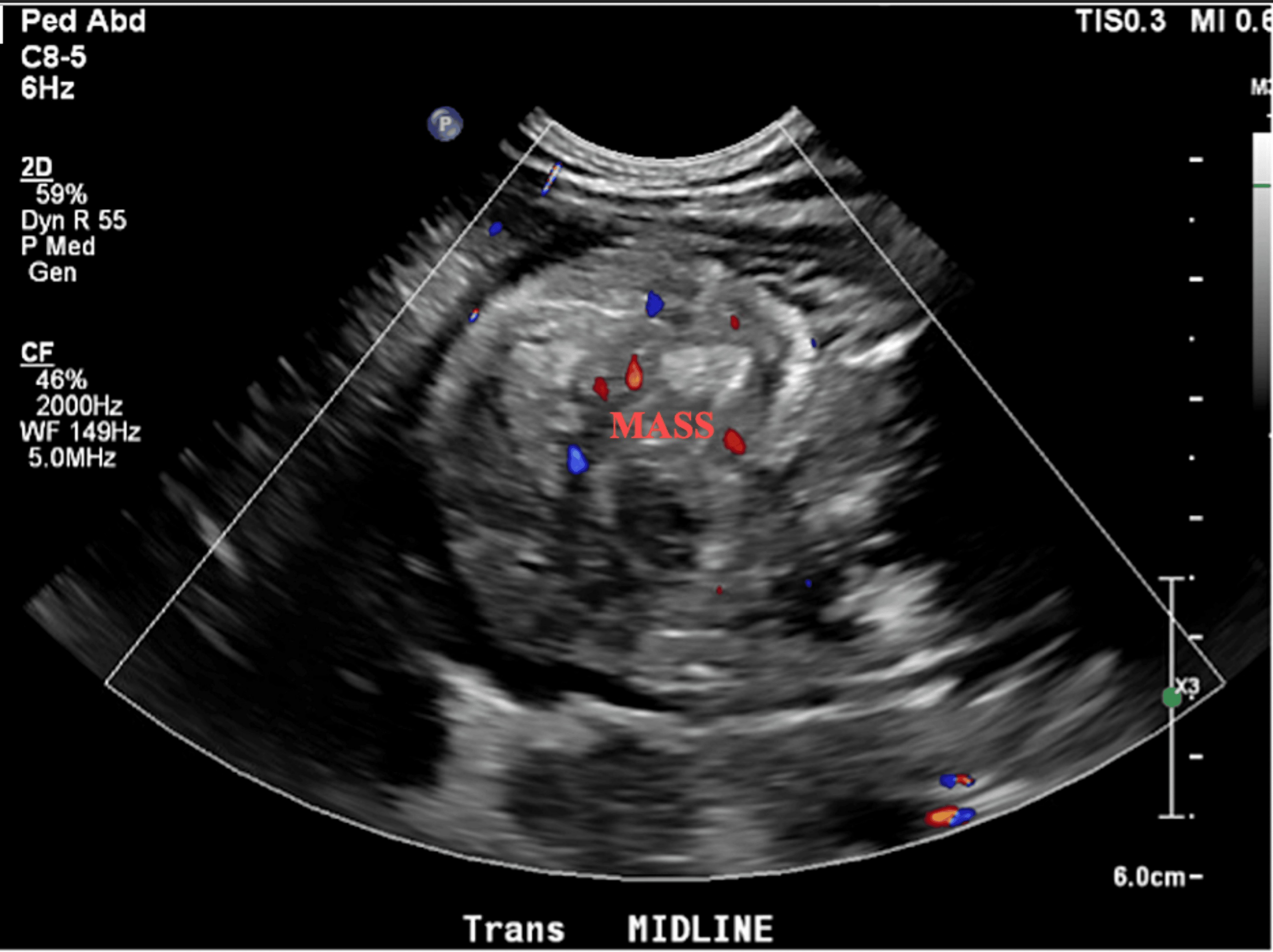
Abdominal ultrasound showing a gastric mass

The initial investigation began with an abdominal ultrasound revealing an echogenic mass that distended the entire stomach, initially thought to be a milk bezoar (Figure 1). This finding prompted further evaluation with esophagogastroduodenoscopy, which then revealed a gastric mass with evidence of bleeding. Partial gastric resection was then performed with Roux-en-Y reconstruction. Pathology studies of the resected specimen confirmed the diagnosis of the mass to be gastric duplication with adjacent benign tumor adenomyomatosis.

Remarkably, the patient’s anemia resolved following the removal of the duplication cyst. This was evidenced at her three-month follow-up after the surgical procedure, at which point the patient had normal hemoglobin (11.8 g/dL), and her reticulocytosis had resolved (corrected reticulocyte 1.58%). Iron studies at this time showed no evidence of iron deficiency anemia. Given the presence of anemia with significant reticulocytosis, as well as the resolution of her symptoms once the cyst was removed, the diagnosis was confirmed to be paraneoplastic autoimmune hemolytic anemia in the setting of gastric duplication with associated adenomyomatosis, a rare phenomenon previously only recorded in two adult case reports.

## Discussion

Our report presents the rare association between an asymptomatic GDC and hemolytic autoimmune anemia in a pediatric patient. This phenomenon occurring in the pediatric population has not been reported in the literature thus far.

Gastric duplication is a rare entity with a clinical picture ranging from asymptomatic to signs of obstruction, peritonitis, or, as seen in our case, anemia of unknown origin. Hemolytic anemia is classically associated with hematologic malignancies, autoimmune disease, infections, drugs, and very rarely ovarian dermoid cysts and mesenteric dermoid cysts. Previous cases reported in the adult population suggest that autoimmune hemolytic anemia occurred as a paraneoplastic syndrome in the setting of adenocarcinoma arising in the cyst [8,9]. Our case is unique in that the paraneoplastic syndrome was due to adenomyomatosis adjacent to the GDC rather than carcinoma. In addition, this case challenges the current belief that gastric duplications tend to remain benign in the pediatric population only to cause malignant transformation much later in life [9].

Diagnosis is difficult given the non-specific presentation of a GDC, and investigation should include radiography, ultrasound, computerized tomography, and magnetic resonance imaging. Diagnosis of a GDC is histopathological and requires the following criteria to be fulfilled: 1) the wall of the cyst is continuous with the stomach wall; 2) the cyst is surrounded by smooth muscle that is continuous with gastric muscle; 3) the cyst shares a common blood supply to the stomach; and 4) the cyst is lined by alimentary epithelium of any type [10].

The correct identification and diagnosis of patients with GDCs is imperative due to the high risk of malignant transformation of these congenital abnormalities. Additional complications include obstruction, torsion, perforation, and hemorrhage [10]. Furthermore, hemolysis has been noted to resolve following complete surgical resection of a GDC, further supporting this as the preferred treatment modality [8]. A minimally invasive laparoscopic approach is used to perform enucleation and partial gastrectomy [3,9]. If multiple organs or structures are involved, more invasive and reconstructive surgery is required, as in the case we reported. Prognosis, therefore, is dependent on the extent of surgery required, recurrence of cysts, and future need for additional surgeries.

## Conclusions

Gastric duplication is an incredibly rare congenital disorder. We report the only case known to us of autoimmune hemolytic anemia as a paraneoplastic syndrome secondary to a GDC in pediatrics. Following the surgical removal of this cyst, the patient’s anemia was completely resolved. This patient had been persistently treated for anemia attributed to other etiologies without improvement. In future cases of young children with anemia of unknown origin, we suggest comprehensive imaging studies be performed to exclude anatomical anomalies such as GDCs.
